# The Effects of Palm Oil on Plasma and Serum Lipid Parameters: A Systematic Review on Animal Intervention Studies

**DOI:** 10.3389/fvets.2020.00303

**Published:** 2020-07-07

**Authors:** Syed-Badrul Syarifah-Noratiqah, Syed Fairus, Mohamed S. Zulfarina, Zafri Nasrullah, H. M. S. Qodriyah, Isa Naina-Mohamed

**Affiliations:** ^1^Department of Pharmacology, Faculty of Medicine, Universiti Kebangsaan Malaysia, Kuala Lumpur, Malaysia; ^2^Metabolics Unit, Advanced Biotechnology and Breeding Centre (ABBC), Malaysian Palm Oil Board (MPOB), Kajang, Malaysia

**Keywords:** dyslipidemia, hyperlipidemia, metabolic syndrome, palm oil, palm olein

## Abstract

**Background:** Accumulative evidences on the beneficial effects of palm oil are progressively reported; however, there are still several controversies related to their effects on the risks of cardiovascular disease (CVD). This review explores the effects of palm oil and its liquid fraction namely palm olein, which is commonly used as cooking oil on four lipid parameters; total cholesterol (TC), triglyceride (TG), high-density lipoprotein-cholesterol (HDL-C) and low-density lipoprotein-cholesterol (LDL-C), which play an important role as CVD-related biomarkers. A systematic review of the literature was conducted to identify the relevant studies on palm oil and the lipid parameters specifically focusing on the *in-vivo* animal model.

**Methods:** A comprehensive search was conducted in Medline *via* EBSCOhost, Medline *via* OVID and Scopus. Studies were limited to the English language published between the years of 2000 and 2019. The main inclusion criteria were as follows: (1) Study with *in-vivo* animal experiments [the animal should be limited to mammals] (2) Study should have evaluated the effects of palm oil or palm olein on plasma or serum lipid parameter (3) Study should have used palm oil or palm olein in the form of pure or refined oil (4) The treatment of palm oil or palm olein was assessed using the following outcomes: plasma or serum TC, TG, HDL-C, and LDL-C concentration (5) Study should have control group and (6) studies on specific fatty acid, fraction enriched tocotrienol and tocopherol, crude palm oil, kernel oil, red palm oil, thermally oxidized palm oil, hydrogenated palm oil, and palm oil or palm olein based products namely margarine, palm milk, butter and cream were excluded. The quality and the risk of bias on the selected studies were assessed using the ARRIVE Guideline and SYRCLE's Risk of Bias tools, respectively.

**Results:** The literature search successfully identified 17 potentially relevant articles, whereby nine of them met the inclusion criteria. All research articles included in this review were *in vivo* studies comprising seven rats, one hamster and one mice model.

**Conclusion:** Significant positive outcomes were observed in several lipid parameters such as TC and LDL-C. The evidence from this review supported that palm oil and palm olein possess high potential as lipid-lowering agents.

## Introduction

The application of vegetable oils for daily food consumption and manufacturing of health supplement products is increasing. Over the years, the global demand for palm oil (PO) has rapidly increased for edible and non-edible purposes (1). The oil palm tree (*Elaeis guineensis*) provides the highest yield of oil when properly cultivated compared to other oil-seed crops. This high oil-producing agricultural crop that belongs to the *Palmae* or *Arecaceae* family (2) has an economic lifespan of 20–25 years (3). The oil palm oil tree is cultivated in 43 countries worldwide, among which Indonesia, followed by Malaysia, are the world's largest producers of PO (4).

There are two distinct oils extracted from the fruits of the oil palm tree: PO and palm kernel oil (PKO) (1, 2). The crude palm oil (CPO) is the primary oil derived from the fleshy orange-red mesocarp of the oil palm fruits and is rich in palmitic acid. The edible CPO, which is in a semi-sold form at the room temperature, undergoes refining, and fractionation processes to form the liquid fraction of palm olein and solid fraction of palm stearin (1, 5). Palm olein that remains completely liquid at room temperature is commonly bottled and sold as cooking oil. On the other hand, the solid form of palm stearin is widely used in producing vegetable ghee, margarine and shortening (6). Secondary oil derived from oil palm fruits, PKO, is the minor oil extracted from the seed or kernel of the oil palm fruits. In contrast to PO, PKO is high in lauric acid, resembling coconut oil in taste and odor. This light yellow oil is suitable for use in confectioneries, edible fats and baked goods, as well as in manufacturing soaps and detergents (1, 6).

The major components of palmitic acid (C16:0), which account for almost 50% of total fatty acids, have made PO to be classified as a saturated fat group (7). Despite the fact that saturated fat is associated with the increased risk of cardiovascular disease (CVD) (8), PO possesses a distinct effect due to the presence of monounsaturated fatty acids (MUFA) in which 80% are in the sn-2 position of triacylglycerols (TAG) (7). This unsaturated fat, also known as oleic acid (C18:1), is the major fatty acid of olive oil. Several studies have demonstrated the beneficial effects of PO that are similar to olive oil even though it consists of a high amount of saturated fat (9–12). The degree saturation of fatty acids in the sn-2 position plays an important role in determining the atherogenicity of specific fatty acids (13). In addition, PO is cholesterol free and consists only of a small amount of atherogenic saturated fats, namely lauric acid (C12:0), and myristic acid (C14:0) (14).

CVD that comprises heart, brain vascular, and blood vessel diseases is a leading cause of 17.3 million deaths per year (15). Hyperlipidaemia is defined as the elevation of fasting total cholesterol (TC) and/or triglyceride (TG) level, which is recognized as a risk factor of CVD (16). There are four lipid associated particles that play an important role as CVD-related biomarkers, which are TC, TG, high-density lipoprotein cholesterol (HDL-C) and low-density lipoprotein cholesterol (LDL-C). TC is the sum of cholesterol including LDL-C, very low-density lipoprotein cholesterol (VLDL-C), and HDL-C in the serum or plasma blood (17). The LDL-C, which is also known as “bad cholesterol,” is generally measured for predicting CVD that possesses atherogenic properties (18). Elevated LDL-C level in the blood often leads to the development of atherosclerosis and coronary heart disease (19). In contrast to LDL-C, HDL-C is a “good cholesterol” that possesses atheroprotective effects. The elevation of this lipid panel is considered an inverse factor to cardiovascular disease (20). TG is another important lipid panel used for energy storage (21). Similar to TC and LDL-C, a significant elevation of serum or plasma TG concentration is associated with myocardial infarction and atherosclerosis (21–23). Higher concentration of TG in the circulation above normal can lead to hypertriglyceridemia.

Although there is cumulative evidence on the beneficial effects of PO, there are several considerable controversies on their harmful effects, leading to CVD. Thus, this article aims to systemically review the effects of PO and palm olein. Relevant studies on PO, palm olein and four lipid panels (TC, TG, HDL-C, and LDL-C) specifically focusing on the *in-vivo* animal model were identified and carefully selected for the current review. This review has been restricted solely to animal studies, specifically on mammals, to form a solid foundation on the overview conclusion and due to the large number of articles available. A systematic review on the effects of PO and palm olein related to lipid panels in human studies shall be conducted in the near future.

## Materials and Methods

### Literature Search

A systematic review was conducted to identify relevant studies about the effects of PO and palm olein on serum and plasma lipid parameters. Previous reports were retrieved from different electronic databases including Medline *via* EBSCOhost, Medline *via* OVID, and Scopus. Studies were limited to the English language published between year 2000 and 2019. The search strategy involved a combination of the following two sets of keywords: (1) palm^*^ oil^*^ OR palm^*^ olein^*^ OR *Elaeis guineensis* AND (2) ^*^cholesterol^*^ OR ^*^triglyceride^*^ OR ^*^lipoprotein^*^ OR ^*^lipid^*^ OR LDL OR HDL.

### Inclusion and Exclusion Criteria

**Figure 1:**
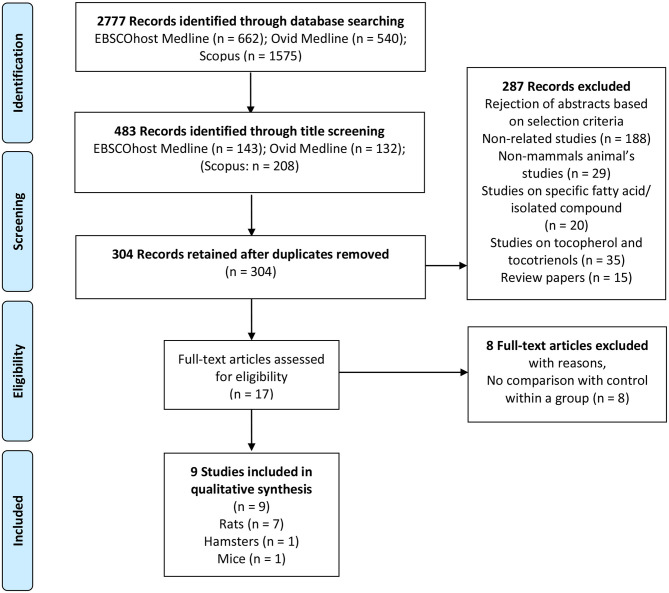
PRISMA flow diagram (animal study). Summary of literature search, screening and selection of potential studies. PRISMA flow diagram represents the literature search in three different electronic data bases, which are EBSCOhost Medline, Ovid Medline, and SCOPUS followed by screening and inclusion of eligible studies for systematic review.

Through the database searches, articles published in the English language including abstracts were selected. Due to the large number of articles searched from Scopus, the results were further limited to the subject area of “Medicine” and “Pharmacology, toxicology, and pharmaceutics.” Research articles that met the inclusion criteria were included for further analyses: (1) Study with *in-vivo* animal experiment [the animal should be mammals, animals such as birds and fish were excluded]; (2) Study should have evaluated the effects of PO or palm olein on plasma or serum lipid parameter; (3) Study should have used PO or palm olein in the form of pure or refined oil; (4) The treatment of PO or palm olein was assessed using the following outcomes: plasma or serum TC, TG, HDL-C, and LDL-C concentration; (5) Study should have a control group; and (6) Studies on specific fatty acids, fraction enriched tocotrienols and tocopherols, crude PO, kernel oil, red PO, thermally oxidized PO, hydrogenated PO, and PO or palm olein based products such as margarine, coconut milk, butter, and cream were excluded. Review articles, case studies, editorials, news and letters were excluded. PRISMA flow diagram for the summary of the literature search, screening and selection of potential studies is presented in Figure 1.

### Selection of Potential Studies

The research studies reporting the effects of PO or palm olein on plasma, or serum lipid parameter in animals were selected based on the title and abstract by two independent reviewers. When the decision could not be finalized through the abstract, full articles were reviewed and evaluated. Reviewers independently evaluated the eligibility of each potential full-text article. Any disagreement or differences in opinions was resolved through the discussion between reviewers.

### Data Extraction and Management

Three phases of screening procedure were conducted before final papers were included in the review: (1) The first phase is the title screening where the papers were excluded if they did not match the inclusion criteria based on the title; (2) The second phase is the abstract screening where the remaining papers were screened based on their abstract, and papers that did not meet the inclusion criteria were thus excluded; (3) The third phase is full-text screening where the remaining papers were read thoroughly to exclude any paper that did not meet the inclusion criteria. Duplicates were removed and the remaining articles were again screened for eligibility by reviewers. Both reviewers should be in an agreement for the articles selected in the review before the data extraction phase. Data extraction was performed independently by the reviewers using a data collection form for standardization. The following data were recorded from the studies: (1) First author with year of publication; (2) A brief description of animal species and the strain used in the study; (3) A brief description of gender, age, and weight of animal used in the study; (4) Group size of animal used in the study; (5) A brief description of the study design; (6) A brief description of the amount or dose of intervention; (7) Duration of intervention and evaluation time point; (8) A brief description of methodology; (9) A brief description of the results; and (10) Outcome measures. The evidence table of the studies selected is tabulated in Table 1.

**Table 1:** The symbol “↑” indicates significantly higher.

	**Species (strain)**	**Gender, age, and weight**	**Group size**	**Study design**	**Amount/dose of intervention**	**Duration of intervention and evaluation time point**	**Methodology**	**Results**	**Outcome measures**
**Study 1**									
(24)	Wistar rats	G: Male A: NA W: 150–200 g	Gr1: 22 Gr2: 22 Gr3: 22 Gr4: 22	Gr1: ND Gr2: ND + 25% hydrogenated PO Gr3: ND + 25% palm olein Gr4: ND + 25% canola oil	25% oils were mixed with ND. ND was made of normal rats chow.	DOI: 4 weeks ETP: Week 4	Serum lipid profile was measured in the form of TC, TG, HDL-C, and LDL-C	Serum TC concentration: palm olein group slightly higher but ≠ than control group Serum TG concentration: palm olein group ↑ than control group Serum HDL-C concentration: palm olein group ↑ than control group Serum LDL-C concentration: palm olein group ↓ than control group	In this study, palm olein is an excellent reducer of serum LDL-C concentration and contributes to the elevation of TG and HDL-C concentration. No changes were seen in serum TC concentration
**Study 2**									
(25)	Sprague-Dawley rats	G: Male A: 7 weeks old W: 309 ± 5.03 g	Gr1: 5 Gr2: 5 Gr3: 5 Gr4: 5	Gr1: ND Gr2: ND + 2.5 ml PO Gr3: ND + 2.5 ml sunflower oil Gr4: ND + 2.5 ml palm and sunflower oil (1:1)	Rats were treated with 2.5 ml of oils per day and fed with purified diet/ND throughout the experimental period.	DOI: 22 days ETP: Day 10 and 22	Serum lipid profile was measured in the form of TC, TG, HDL-C, and LDL-C	Serum TC concentration: PO group ↓ than control group Serum TG concentration: PO group reduced but ≠ than control group Serum HDL-C concentration: PO group ↓ than control group Serum LDL-C concentration: PO group reduced but ≠ than control group	In this study, PO was an excellent reducer of serum TC and HDL-C concentration. Serum TG and LDL-C concentration also reduced but not significant compared to control group
**Study 3**									
(26)	Albino rats	G: Male A: NA W: 90–120 g	Gr1: 6 Gr2: 6 Gr3: 6 Gr4: 6	Gr1: ND Gr2: SD + 35% distilled water Gr3: SD + 35% PO Gr4: ND + 35% PO mill effluent	35% oils were mixed with SD consisting 50% corn starch and 15% soybean powder. Pelletised mouse chow served as control (ND)	DOI: 6 weeks ETP: Week 6	Serum lipid profile was measured in the form of TC, TG, HDL-C, and LDL-C	Serum TC concentration: PO group slightly higher but ≠ than control group and ↑ than lipid-free diet group Serum TG concentration: PO group ≠ than control group and ↑ than lipid-free diet group Serum HDL-C concentration: PO group ≠ than control group and lipid-free diet group Serum LDL-C concentration: PO group ↑ than control group and ≠ than lipid-free diet group	In this study, PO significantly elevated Serum LDL-C concentration and no changes were seen in serum TC, TG and HDL-C concentration
**Study 4**									
(27)	Wistar-Kyoto rats	G: Male A: 3 weeks old W: NA	Gr1: 6 Gr2: 6 Gr3: 6 Gr4: 6	Gr1: ND Gr2: ND + 25% super olein Gr3: ND + 25% red palm olein Gr4: ND + 25% palm olein	25% oils were mixed with ND. ND was made of standard chow diet	DOI: 15 weeks ETP: Week 15	Serum lipid profile was measured in the form of TC, TG, HDL-C, and LDL-C	Serum TC concentration: palm olein group ≠ than control group Serum TG concentration: palm olein group ≠ than control group Serum HDL-C concentration: palm olein group ≠ than control group Serum LDL-C concentration: palm olein group ≠ than control group	In this study, no changes were seen in serum TC, TG, HDL-C, and LDL-C concentration of palm olein group compared to control group
**Study 5**									
(28)	White Albino rats (*Rattus norvegicus*)	G: Male A: NA W: 200–250 g	Gr1: 8 Gr2: 8 Gr3: 8	Gr1: ND Gr2: ND + 10% PO Gr3: ND + 10% karnel oil	10% oils were mixed with ND. ND was made of rat mash	DOI: 35 days ETP: Day 35	Serum lipid profile was measured in the form of TC, TG, HDL-C, and LDL-C	Serum TC concentration: PO group slightly higher but ≠ than control group Serum TG concentration: PO group ↑ than control group Serum HDL-C concentration: PO group ↓ than control group Serum LDL-C concentration: PO group ≠ than control group	In this study, PO significantly elevated Serum TG and reduced LDL-C concentration. No changes were seen in TC and LDL-C concentration of PO group compared to control group
**Study 6**									
(29)	Swiss mice	G: Male A: NA W: 25 ± 5 g	Gr1: 5 Gr2: 5 Gr3: 5 Gr4: 5 Gr5: 5 Gr6: 5 Gr7: 5 Gr8: 5 Gr9: 5	Gr1: ND Gr2: ND + 7% soybean oil Gr3: ND + 7% corn oil Gr4: ND + 7% PO Gr5: ND + 7% olive oil Gr6: ND + 7% sunflower oil Gr7: ND + 7% butter Gr8: ND + 7% animal fat Gr9: ND + 7% margarine	7% oils were mixed with ND. ND was made of basal diet (AIN-93G)	DOI: 6 weeks ETP: week 6	Serum lipid profile was measured in the form of TC, TG, HDL-C, and LDL-C	Serum TC concentration: PO group ≠ than control group Serum TG concentration: PO group ↓ than control group Serum HDL-C concentration: PO group ↓ than control group Serum LDL-C concentration: PO group ↓ than control group	In this study, PO was an excellent reducer of serum TG, HDL-C, and LDL-C concentration. Serum TC concentration also reduced but not significant compared to control group
**Study 7**									
(30)	Albino Rats	G: Male A: NA W: NA	Gr1: 5 Gr2: 5 Gr3: 5 Gr4: 5	Gr1: ND + 1 ml crude PO Gr2: ND + 1 ml refined palm olein Gr3: ND + 1 ml crude groundnut oil Gr4: ND	Rats were treated with 1 ml of oils per day and fed with ND throughout the experimental period.	DOI: 28 days ETP: Day 28	Plasma lipid profile was measured in the form of TC, TG, HDL-C, and LDL-C	Plasma TC concentration: palm olein group ↓ than control group Plasma TG concentration: palm olein group ↑ than control group Plasma HDL-C concentration: palm olein group lower but ≠ than control group Plasma LDL-C concentration: palm olein group ↓ than control group	In this study, refined palm olein was an excellent reducer of plasma TC and LDL-C concentration. Refined palm olein significantly elevated plasma TG concentration and no changes were seen in HDL-C concentration of refined palm olein group compared to control group
**Study 8**									
(31)	Rats (Balthazar)	G: Male A: 30 days old W: 149 ± 1 g	Gr1: 4 Gr2: 4	Gr1: ND + 12% Palm olein Gr2: ND	12% oils were mixed with ND.	DOI: 60 days ETP: day 60	Serum lipid profile was measured in the form of TC, TG, HDL-C, and LDL-C	Plasma TC concentration: PO group slightly higher but ≠ than control group Plasma TG concentration: PO group ≠ than control group Plasma HDL-C concentration: PO group ↑ than control group Plasma LDL-C concentration: PO group slightly lower but ≠ than control group	In this study, PO significantly elevate serum HDL-C concentration and no changes was seen in TC, TG and LDL-C concentration of PO group compared to control group
**Study 9**									
(32)	F_1_B Golden Syrian hamsters (*Mesocricetus auratus*)	G: NA A: NA W: NA	Gr1: 12 Gr2: 12 Gr3: 12 Gr4: 12	Hamsters were fed with 10% CO and 0.1% cholesterol to establish hyperlipidaemia for 2 weeks. CO group was continued as hyperlipidaemic model for 10 weeks. All other groups were treated with different type of oils as below after 2 weeks of establishment of hyperlipidaemia Gr1: ND + 10% coconut oil Gr2: ND + 10% red PO Gr3: ND + 10% RBD-PO Gr4: ND + reconstituted RBD-PO	10% oils were mixed with ND. ND was made of non-purified diet	DOI: 10 weeks ETP: week 8 and 10	Plasma lipid profile was measured in the form of TC, TG, HDL-C, and LDL-C	Plasma TC concentration: RBD-PO group ↓ than control group Plasma TG concentration: RBD-PO group ↓ than control group Plasma HDL-C concentration: RBD-PO group ↑ than control group Plasma non-HDL-C concentration: RBD-PO group ↓ than control group	In this study, refined, bleached and deodorized PO was an excellent reducer of plasma TC, TG and non-HDL-C concentration. RBD-PO significantly elevate plasma HDL-C concentration

*The symbol “↓” indicates significantly lower. The symbol “≠” indicates no significant difference. CO, coconut oil; HDL-C, high density lipoprotein cholesterol; LDL-C, low density lipoprotein cholesterol; NA, not available; ND, normal diet; PO, palm oil; RBD-PO, refined, bleached, and deodorized palm oil; SD, special diet; TC, total cholesterol; TG, triglyceride*.

### Assessing the Quality of the Studies

Nine potential studies were assessed by the reviewers for methodological quality using the Animal Research: Reporting of *in-vivo* Experiment (ARRIVE) Guidelines to improve the reporting of research using animals (33). This guideline, which has been developed using the CONSORT statement as their foundation, consists of a checklist of 20 items describing the minimum information that all scientific publications reporting research using animals should include (34–36). The quality of the studies selected was assessed based on the percentage of the individual items in the ARRIVE Guidelines checklist as per displayed in Table 2.

**Table 2:** The quality assessment of studies included (ARRIVE guidelines).

**Recommendation**	**Item no**.	**Studies**									**Percentage of the studies that met the criteria of the ARRIVE guidelines (%)**
		**Amini et al. (24)**	**Go et al. (25)**	**Ajiboye et al. (26)**	**Boon et al. (27)**	**Ibegbulem and Chikezie (28)**	**Rezq et al. (29)**	**Badmus et al. (30)**	**Karaji-Bani et al. (31)**	**Wilson et al. (32)**	
**Title**											
Accurate and concise	1	√	√	√	√	√	√	√	√	√	100%
**Abstract**											
Summary of the background, research objectives, details of the species or strain of animal used, key methods, principal findings, and conclusions	2	√	√	√	√	√	√	√	√	√	100%
**Introduction**											
Background—sufficient scientific background	3a	√	√	√	√	√	√	√	√	√	100%
Background—explanation of the animal model selected relevance to human biology	3b	X	X	X	√	X	X	X	X	√	22%
Objectives—primary and any secondary objectives, or specific hypotheses	4	√	√	√	√	√	√	√	√	√	100%
**Methods**											
Ethical statement—ethical review permissions, relevant licenses, and national or institutional guidelines for the care and use of animals	5	X	√	√	√	X	X	X	X	√	44%
Study design—the experimental number and control groups	6a	√	√	√	√	√	√	√	√	√	100%
Study design—steps taken to minimize the effects of subjective bias, when allocate animal to treatment and assessing results	6b	√	√	√	√	√	√	√	√	√	100%
Study design—experimental unit	6c	√	√	√	√	√	√	√	√	√	100%
Experimental procedures—precise details of how the procedure is carried out	7a	√	√	√	√	√	√	√	√	√	100%
Experimental procedures—precise details of when the procedure is carried out	7b	√	√	√	√	√	√	√	X	√	89%
Experimental procedures—precise details of where the procedure is carried out	7c	√	√	X	√	√	√	X	√	√	78%
Experimental procedures—precise details of why the procedure carried out	7d	√	√	√	√	√	√	X	X	√	78%
Experimental animals—details of the animals used, species, strain, sex, developmental stage, and weight	8a	√	√	√	√	√	√	X	√	√	89%
Experimental animals—relevant information such as source of animals and international strain nomenclature	8b	X	√	X	√	√	√	√	X	√	67%
Housing and husbandry—housing	9a	X	√	X	√	√	√	X	√	√	67%
Housing and husbandry—husbandry conditions	9b	√	√	√	√	√	√	X	√	√	89%
Housing and husbandry—welfare-related assessments and interventions	9c	X	√	√	√	√	X	√	√	√	78%
Sample size—total number of animals used in the experiment and number of animals in each group	10a	√	√	√	√	√	√	√	√	√	100%
Sample size—determination of the animal numbers and details of any sample size calculation used	10b	√	X	X	X	X	X	X	X	X	11%
Sample size—number of independent replications, if relevant	10c	X	X	X	X	X	X	X	X	X	0%
Allocating animals to experimental group—details of allocation of the animal to experimental group (randomization or matching)	11a	√	√	√	√	√	√	√	√	√	100%
Allocating animals to experimental group—order of animals treated and assessed	11b	√	√	√	√	√	√	√	√	√	100%
Experimental outcomes—primary and secondary experimental outcomes	12	√	√	√	√	√	√	√	√	√	100%
Statistical methods—details of statistical methods used	13a	√	√	√	√	√	√	√	√	√	100%
Statistical methods—unit of analysis for each dataset	13b	√	√	√	√	√	√	√	√	√	100%
Statistical methods—any methods used to assess whether or not the data met the assumptions of the statistical approach	13c	√	√	√	√	√	√	√	√	√	100%
**Results**											
Baseline data—relevant characteristics and health status of animals	14	X	√	X	√	√	X	X	√	√	56%
Numbers analyzed—number of animals in each group included	15a	X	X	√	√	√	X	√	X	√	56%
Numbers analyzed—reasons if any animals or data were not included in the analysis	15b	X	X	X	X	X	X	X	X	X	0%
Outcomes and estimation—results for each analysis carried out with measure of precision	16	√	√	√	√	√	√	√	√	√	100%
Adverse events—details of all important adverse events	17a	X	X	X	X	X	X	X	X	X	0%
Adverse events—modifications to the experimental protocols made to reduce adverse events	17b	X	X	X	X	X	X	X	X	X	0%
**Discussion**											
Interpretation/scientific implications—interpretation of the results (study objectives and hypotheses, current theory and other relevant studies)	18a	√	√	√	√	√	√	√	√	√	100%
Interpretation/scientific implications—study limitations (potential source of bias, limitations of animal model and imprecision of the results)	18b	X	X	X	X	X	X	X	X	X	0%
Interpretation/scientific implications—implications of the experimental methods or finding for the replacement, refinement, and reduction (the 3Rs) of the use of animals	18c	X	X	X	X	X	X	X	X	X	0%
Generalisability/translation—comment on how the findings of the study can be translated to other systems including any relevance to human biology	19	√	X	√	√	X	X	√	√	√	67%
Funding—lists all funding sources and the role of the funder(s)	20	√	√	X	√	X	X	X	X	√	44%

“*√” sign Indicates that the criteria of the studies selected have met the ARRIVE guidelines. “X” sign indicates that the criteria of the studies selected did not met the ARRIVE guidelines. The total number of studies that met the criteria of ARRIVE guidelines were presented in the form of percentage. The checklist of 20 items in the recommendation part is followed by the ARRIVE guidelines by Kilkenny et al. (33)*.

### Assessing the Risk of Bias of the Studies

**Figure 2:**
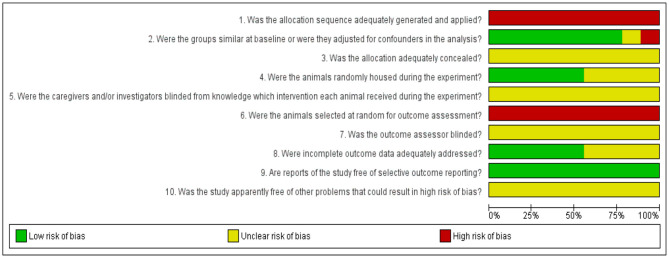
Results of the risk of bias assessment of the nine studies included in this systematic review. Ten items were utilized to assess study quality by scoring reporting. A “yes” score indicates a low risk of bias (green), “no” score indicates a high risk of bias (red) and “unclear” score indicates an unknown risk of bias (yellow). The checklist of 10 items is followed by the SYRCLE's Risk of Bias tool (37).

Risk of bias of the included studies was assessed by the reviewers using SYRCLE's Risk of Bias tool (37). This review focused on five different types of bias, which included selection bias (items 1, 2, and 3), performance bias (items 4 and 5), detection bias (items 6 and 7), attrition bias (item 8), reporting bias (item 9), and other sources of bias (item 10). Risk of bias graph for the studies selected was established with the assistance of RevMan5.3 (Cochrane Library) software as presented in Figure 2. The judgments of “yes” denoting low risk of bias (green), “no” denoting high risk of bias (red) and “unclear” denoting unknown risk of bias (yellow), were reported to properly assess the risk of bias.

## Results

### Search Results

Two thousand seven hundred and seventy-seven articles were assessed from Medline via EBSCOhost, Medline *via* OVID, and Scopus databases. Two reviewers independently assessed all articles for inclusion and exclusion criteria as previously mentioned in section Inclusion and Exclusion Criteria. Two thousand two hundred and ninety-four articles with inadequate thematic were excluded after their titles were read and 179 duplicated studies were removed. Three hundred and four abstracts were read for further selection and 287 studies were excluded since they did not fulfill the inclusion criteria. The rejection of abstracts was based on these selection criteria: (1) 188 articles were non-related studies; (2) 29 articles were non-mammals animal's studies; (3) 20 articles were studies on specific fatty acid or isolated compounds; (4) 35 articles were studies on tocopherol and tocotrienols; and (5) 15 articles were review papers. The remaining 17 full-text articles were assessed for eligibility. Eight full-text articles with no control within a group were excluded. The remaining nine articles that fulfilled all inclusion and exclusion criteria were retrieved for further assessment and data extraction. The flow chart for the summary of a literature search, screening, and selection of potential studies, including reasons for exclusion, is presented in Figure 1.

### Characteristics of the Included Studies

Table 1 displays the summary of the characteristics of all studies. All the articles selected were *in vivo* animal studies published between years 2000 and 2019. The experimental models used in these studies consisted of seven rats, one hamster, and one mice model. Rat is the most frequently used experimental model and the main strains cited were Wistar rats (24, 27); Sprague Dawley rats (25); and Albino rats (26, 28, 30). Regarding animal's gender, all the studies used male rats as the experimental model. Out of seven rat model studies, one study mentioned only the age of the rats (27), three studies mentioned the weight of the rats (24, 26, 28), two studies mentioned the age and weight of the rats (25, 31) and another one study did not mention the age and weight of the rats (30). The number of rats for each group of the study ranged from 5 to 22 rats per group. As for the other two animal models selected, which were mice and hamsters, the species and strain cited were Swiss mice (29) and F1B Golden Syrian hamsters (*Mesocricetus auratus*) (32), respectively. The gender of the mice used in the study was male and the study only mentioned the weight of the mice selected. There was no information on the age of mice, and the number of mice in each group of the study was five mice per group. The third animal model selected for this review was hamster. Forty-eight F1B hamsters were selected for the study, which was further divided into four groups with 12 hamsters per group. The gender, age, and weight of the hamsters were not indicated in the study.

All studies were conducted using an experimental design which compared the outcomes of PO or palm olein groups with the control within the group. Out of nine studies, seven studies formulated a diet by supplementing PO or palm olein in the range of 7–35%. Another two studies conducted showed that the animals were provided with 1 mL of palm olein per day (route of administration was not mentioned) (30) and 2.5 mL of PO per day given through the oral administration (25). The majority of the studies used serum [(24–27), (28, 29, 31)] for the lipid profile analysis. All studies measured four lipid variables (TC, TG, HDL-C, and LDL-C) in their lipid profile study.

### Quality Assessment of the Studies

Table 2 presents the percentage of the individual items in the ARRIVE Guidelines checklist to assess the quality of the studies selected in this review. The results showed that all the selected studies provided an accurate title (100%) and abstract with the summary of the background, research objectives, methods, principal findings and conclusions (100%). Regarding the introduction part, most studies included a sufficient scientific background (100%) and clearly described the study objectives or specific hypotheses being tested (100%). Only several studies explained the reasons for the animal model being used, which addressed the scientific objectives and relevance to human biology (22%).

Only 44% of the studies provided ethical statements for the care and use of animals. All the studies reported the details of the study design, including the number of experimental and control groups (100%), steps taken when allocating animals to treatment (100%), and the experimental unit (100%). For experimental procedures, 100% of the selected studies provided precise details on the dose of intervention. Eighty nine percentage of studies clearly indicated the intervention interval, 78% of the studies provided the information on where the animals were placed during the experiment, and 78% of studies clarified the justification for selecting a specific number of interventions. For the experimental animals, 89% of the selected studies reported the details of the animals used, species, strain, sex, developmental stage, and weight. Sixty seven percentage of the studies provided further information on the source of animals. Sixty seven percentage of studies provided information on the facilities used for the housing of the animals, 89% stated husbandry conditions such as light/dark cycle and temperature, whereas 78% mentioned welfare-related assessments and interventions. With regard to the sample size, 100% of the studies specified the total number of animals per group involved, 11% of the studies provided the details of sample size calculation and no studies (0%) indicated the number of independent replications of their experiment. In the allocation of the animals to experimental groups, all studies provided full details on how animals were allocated to experimental groups (100%) and described the order where animals in the different experimental groups were treated and assessed (100%). All selected studies clearly defined the primary and secondary experimental outcomes. As for statistical method, 100% of the studies provided comprehensive details of their statistical methods.

Fifty six percentage of the studies reported relevant characteristics and the health status of animals prior to the treatment, and 56% reported the number of animals per group included in each analysis. No studies however mentioned if any animals or data were excluded. As for the outcomes and estimation, all studies reported the results for each analysis with measures of precision (100%). No studies provided details on adverse events (0%) and described any modifications to the experimental protocols to reduce adverse events (0%).

For the interpretation or scientific implications in the discussion section, all of the studies interpreted the results based on the study objectives and hypotheses, including the current theory and other relevant studies in the literature. No studies commented on the study limitations, including any potential sources of bias, animal model, and the imprecision associated with the results (0%), nor described any implications in experimental methods or findings for the replacement, refinement or reduction (the 3Rs) of the use of animals in research (0%). Sixty seven percentage of the studies discussed the translation of their current findings to other species, including humans. Only 44% listed their funding sources (grant number) and the role of the funder.

### Risk of Bias Assessment

The results for the risk of bias assessment of all included studies are listed in Figure 2. As for selection bias, allocation sequence of the animals was randomly assigned into the group (Item 1) and not referred to a random number table, nor using a computer random number generator (high risk of bias). Baseline characteristics were highly reported (78%), which indicates a low risk of bias. The distribution of baseline characteristics such as age and weight were balanced for intervention and control groups (Item 2). All authors poorly described whether or not the allocation of the animal to the different groups had been concealed (Item 3), leading to an unclear risk of bias. For performance bias (items 4 and 5), 56% of the studies mentioned the use of randomization during the placement of the animal into facilities (low risk of bias) and all studies poorly reported the blinding of the experiments to the caregivers and/or investigators (unclear risk of bias). None of the studies reported the random selection for outcome assessment (Item 6) in referring to a random number table or using a computer random number generator (high risk of bias). All studies poorly described the blinding of the experiments to the outcome assessor (Item 7) leading to the unclear risk of bias. For attrition bias (Item 8), 56% of the studies described that all animals were included in the analysis throughout the experiment (Item 8). On reporting bias (Item 9), all studies (100%) clearly profiled their study protocol and reported primary and secondary outcomes (low risk of bias). Finally, other sources of bias were not clearly described, and there was unclear risk of bias for all selected studies. Refer the Supplementary Material for the risk of bias assessment.

## Effects of Palm Olein Against Individual Plasma and Serum Lipid Parameters

### Effects of Palm Oil or Palm Olein Against Total Cholesterol (TC)

A total of nine studies selected for this review measured TC as one of the serum or plasma lipid variables. Out of the nine studies, in comparison to the control group, three studies reported a significant reduction of serum or plasma TC concentration in the group treated with PO or palm olein for a minimum of 22 days to a maximum of 10 weeks treatment (25, 30, 32). Seven studies reported non-significant changes of serum or plasma TC in the PO or palm olein treatment group compared to the control group where the time intervention was within the range of 60 days to 15 weeks of treatment (24, 26–29, 31). No study reported a significant increment of serum or plasma TC in the treatment group.

### Effects of Palm Oil or Palm Olein Against Triglyceride (TG)

Out of the nine studies, two studies reported a significant reduction of serum or plasma TG in the PO or palm olein treatment compared to the control group, with time intervention ranging from 6 to 10 weeks of treatment (29, 32). Four studies reported non-significant changes of serum or plasma TG after PO or palm olein treatment compared to the control group, with the time intervention ranging from 22 days to 15 weeks of treatment (25–27, 31). However, three studies reported a significant increment of serum or plasma TG level in the treatment group when compared to the control group within 28–35 days of treatment (24, 28, 30).

### Effects of Palm Oil or Palm Olein Against High Density Lipoprotein Cholesterol (HDL-C)

Out of the nine studies, three studies reported a significant elevation of serum or plasma HDL-C level in the group treated with PO or palm olein compared to control group, with time intervention ranging from 4 to 10 weeks of treatment (24, 31, 32). Three studies reported non-significant changes in serum or plasma HDL-C level after PO or palm olein treatment compared to control group, with time intervention ranging from 6 to 15 weeks of treatment (26, 27, 30). Further, three studies reported a significant reduction of serum or plasma HDL-C in the treatment group compared to control group within 22 days to 6 weeks of treatment (25, 28, 29).

### Effects of Palm Oil or Palm Olein Against Low Density Lipoprotein Cholesterol (LDL-C)

Out of the nine studies, three studies reported significant reduction of serum or plasma LDL-C concentration in the group treated with PO or palm olein compared to control group, with the time intervention ranging from 4 to 6 weeks of treatment (24, 29, 30). One study reported a significant reduction in LDL-C and VLDL-C in 10 weeks treatment (32). Four studies reported no significant changes in serum or plasma LDL-C after PO or palm olein treatment compared to control group, with the time intervention between 22 days and 15 weeks of treatment (25, 27, 28, 31). One study reported a significant increment of serum or plasma LDL-C level in the treatment group compared to control group after 6 weeks of treatment.

## Discussion

This present review reported a systematic search and data selection of the current literature on the effects of PO and palm olein on serum or plasma lipid in *in-vivo* experimental animal models. The effects of PO and palm olein on the concentration of serum or plasma lipid variables were examined on three different rodent models selected. Four important outcome measures were assessed, namely serum or plasma TC, TG, HDL-C, and LDL-C, by measuring the serum or plasma lipid profile test. These lipid panels were used as a predictor to CVD (38). For all serum or plasma lipid parameters, the beneficial findings or unfavorable effects of PO and palm olein were observed through the pool of nine studies selected.

TC is the sum of cholesterol that includes the LDL-C, VLDL-C, and HDL-C in the serum or plasma blood and the rise of this lipid panel above normal range will increase CVD risk (17). A data pool from six studies reported the non-significant changes of serum or plasma TC of PO and palm olein group compared to control group (24, 26–29, 31). No changes of TC were observed in these six studies, indicating that PO and palm olein are safe for consumption and do not possess any harmful effects. The range of PO and palm olein mixed into the diet was as low as 7% (29) and as high as 35% (26).

In addition, another three studies reported a significant positive effect of PO and palm olein in reducing serum or plasma TC concentration (25, 30, 32), therefore strongly supporting safe consumption. Out of three studies, the mode of administration in two studies (25, 30) were through daily feeding of 2.5 and 1 mL oil, respectively. The precision and accuracy of dose would be higher through the gavage (oesophageal or gastric) and direct feeding compared to the mixing of substances in water or food (39). Whereas, the study conducted by Wilson et al. (32) reported a significant reduction of TC through the incorporation of 10% PO into the diet. The study is comparable with one of the studies conducted by Ibegbulem et al. (28) that also mixed the diet with 10% PO; however, no effect of PO was observed in the serum or plasma TC concentration. Following comparison, the study design by Wilson et al. possessed a longer duration of intervention, larger number of animals per group tested, and Golden Syrian hamster was selected as the animal model. Unlike other rodent models, hamster was the closest unmodified rodent model to humans in terms of circulating lipid associated particles, and is more sensitive to high fat and high cholesterol diet (40). Consumption of PO and palm olein did not give any adverse effects toward the lipoprotein profiles, as no study reported significant increment of serum or plasma TC concentration compared to control group through the pool of nine studies selected.

The elevation of serum or plasma TG concentration above a normal range will lead to hypertriglyceridemia, which will eventually increase the risk to CVD such as myocardial infarction and atherosclerosis (21–23). A pool data from four studies reported insignificant changes of TG compared to control group, which indicates the safe consumption of PO and palm olein (25–27, 31). Another two studies supported the safe consumption of PO and palm olein through the significant reduction of TG, which reflects the potential of this oil in treating hypertriglyceridemia (29, 32). Both studies demonstrated the incorporation 7–10% of oil into the diet. This study reflects the potential of PO or palm olein as a remedy in preventing hypertriglyceridemia if it is consumed in low amount. In addition, another three studies reported the significant increment of serum or plasma TG concentration compared to that of the control group (24, 28, 30). In comparison to the previous six studies, the duration of intervention for the three studies was shorter, which was only up to five weeks. Meanwhile, the duration of intervention for six studies that showed no changes and significantly reduced serum or plasma TG level group was longer, which was up to 15 weeks. Through the trend of data presented here, to fully understand the effects of PO or palm olein on serum or plasma TG level, further studies are required with a low amount of oil mix into the diet and a longer period of dietary intervention.

HDL-C possesses an atheroprotective effect and its elevation is considered as an inverse factor to CVD risk (20). Based on the pool of nine studies selected, three studies reported a significant increment of HDL-C (24, 31, 32), another three studies reported a significant reduction of HDL-C (25, 28, 29), while the remaining three studies reported no changes (26, 27, 30) after PO or palm olein treatment. The pool of this data is not enough to draw a conclusion, since the results of all the studies were evenly distributed.

Over the decades, LDL-C was the most assessed lipid parameter used for predicting CVD (18). The elevation of this atherogenic lipoprotein subfraction often leads to the development of atherosclerosis and coronary heart disease (19). Out of the nine studies, in comparison to the control group, four studies reported no changes on serum or plasma LDL-C concentration following the consumption of PO or palm olein (25, 27, 28, 31). The same number of studies demonstrated the ability of PO or palm olein to significantly reduce the serum or plasma LDL-C concentration (24, 29, 30, 32). These two pools of data demonstrated a significant finding on the safe consumption of this oil, as well as a potential agent to reduce the concentration of LDL-C. It is important to note that out of the nine studies, only one study reported unfavorable effects of the PO on serum or plasma LDL-C concentration after 6 weeks of consumption, in which the amount of oil incorporated into the diet was the highest (35%) among other studies selected in this review (26). Despite the significant increment in serum LDL-C concentration, Ajiboye et al. reported no changes in other lipid variables such as TC, TG and HDL-C compared to the control group. Thus, this data supported the safe consumption of PO or palm olein.

## Limitations and Recommendations

To the best of our knowledge, this is the first evidence-based review that focuses on the effects of PO and palm olein on four lipid panels, which are TC, TG, HDL-C, and LDL-C. Although the PO and palm olein have revealed the safety of consumption as well as capability in improving the blood lipid level, a number of unavoidable limitations were identified in this review. A systematic review of PO and palm olein was conducted on an animal model to study the effects of these oils on serum or plasma lipid panel. A systematic search identified nine relevant research articles thus far. Compared to clinical trials, animal research by nature is more heterogeneous due to the different types of animal, species, and study designs of the included studies. However, the inclusion and exclusion criteria were clearly defined to minimize the heterogeneity among the studies selected. In addition, many studies used a different dose and amount of oils, which led to non-conformity of outcomes and may influence the interpretation of the results. This study search was limited to only studies published in English and current articles published between the years of 2000 and 2019, while there may also be relevant studies written in other languages and published before the year of 2000. In addition, the bias assessment of the included studies was high, specifically on the random outcome assessment and sequence generation.

Based on the results of the lipid panels examined in this review, it is recommended to conduct more studies in the future with a suitable rodent model. Hamster is recommended to be used since it is an established model in the study of plasma lipid, being closer to the human lipoprotein profile. Previous data reported the safe consumption of the oil even at high concentration; however, a small to moderate amount of oil is recommended to be used. While comparing the two modes of administration, direct consumption through oral feeding is recommended as it is more accurate compared to mixing the oil into the diets. Dose exploration is strongly suggested for treatment purposes. In addition, a large number of animals per group and a longer time period for dietary intervention is advisable.

## Conclusion

In conclusion, the evidence from the review suggested that PO or palm olein is safe for general consumption and may possess lipid-lowering properties. Although there are a few studies that reported increasing TG level through the consumption of PO and palm olein, the number of studies that demonstrated safe consumption and positive outcomes were larger. Based on the pool data of the selected studies, the consumption of PO or palm olein does not give any adverse effects and have showed significant positive outcomes toward the circulating TC and LDL-C levels.

## Author Contributions

S-BS-N, MZ, and ZN performed a literature search and drafted the manuscript. SF, HQ, and IN-M provided a critical review for the manuscript. IN-M gave final approval for the publication of this manuscript. All authors contributed to the article and approved the submitted version.

## Conflict of Interest

The authors declare that the research was conducted in the absence of any commercial or financial relationships that could be construed as a potential conflict of interest.
