# Supplementary material for: The Effects of Palm Oil on Plasma and Serum Lipid Parameters: A Systematic Review on Animal Intervention Studies
Source: Front Vet Sci. 2020 Jul 7;7:303. doi: 10.3389/fvets.2020.00303 (PMC7381326; doi:10.3389/fvets.2020.00303)

Supplementary Material

# SYRCLE’s tool for assessing risk of bias

| **Study** | **1** | **2** | **3** | **4** | **5** | **6** | **7** | **8** | **9** | **10** |
| --- | --- | --- | --- | --- | --- | --- | --- | --- | --- | --- |
|  | **Selection bias 1** | **Selection bias 2** | **Selection bias 3** | **Performance bias 1** | **Performance bias 2** | **Detection bias 1** | **Detection bias 2** | **Attrition bias** | **Reporting bias** | **Other potential bias** |
|  | Sequence generation | Baseline characteristics | Allocation concealment | Random housing | Blinding | Random outcome assessment | Blinding | Incomplete outcome data | Selective outcome reporting | Other source of bias |
| Amini *et al*., 2017 [1] | X | √ | ? | √ | ? | x | ? | ? | √ | ? |
| Go *et al*., 2015 [2] | X | √ | ? | ? | ? | x | ? | ? | √ | ? |
| Ajiboye *et al*., 2015 [3] | X | √ | ? | √ | ? | x | ? | √ | √ | ? |
| Boon *et al*., 2013 [4] | X | √ | ? | √ | ? | x | ? | √ | √ | ? |
| Ibegbulem & Chikezie 2012 [5] | X | √ | ? | √ | ? | x | ? | √ | √ | ? |
| Rezq *et al*., 2010 [6] | X | √ | ? | ? | ? | x | ? | ? | √ | ? |
| Badmus *et al*., 2008 [7] | X | ? | ? | √ | ? | x | ? | √ | √ | ? |
| Karaji-Bani *et al*., 2006 [8] | X | √ | ? | ? | ? | x | ? | ? | √ | ? |
| Wilson *et al*., 2005 [9] | X | X | ? | ? | ? | x | ? | √ | √ | ? |


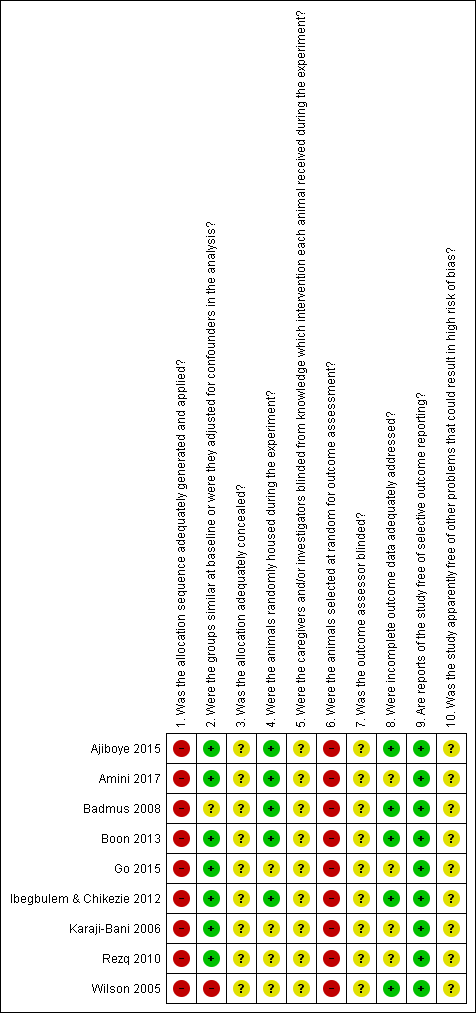

Supplement: Supplementary file 1 [file Data_Sheet_1.docx]
